# Incidence and predictors of multimorbidity among older adults in Korea: a 10-year cohort study

**DOI:** 10.1186/s12877-022-03250-w

**Published:** 2022-07-07

**Authors:** Tae Wha Lee, Jane Chung, Kijun Song, Eunkyung Kim

**Affiliations:** 1 College of Nursing, Mo-Im Kim Nursing Research Institute, Yonsei University, Seoul, Republic of Korea; 2 School of Nursing, Virginia Commonwealth University, Virginia, USA; 3 College of Nursing and Brain Korea 21 FOUR Project, Yonsei University, Seoul, Republic of Korea

**Keywords:** Older adults, Multimorbidity, Predictors, Incidence, Korean longitudinal study of aging, KLoSA, Secondary data analysis, Longitudinal study

## Abstract

**Background:**

Due to the rapid growth of the older adult population, multimorbidity has become a global concern for an aging society. Multimorbidity has been associated with poor health outcomes, including low quality of life and a high risk of mortality, resulting in an overload of healthcare systems. However, multimorbidity incidence and its related factors are poorly understood among older adults. This study aimed to determine whether sociodemographic characteristics, lifestyle, and psychosocial factors predict multimorbidity incidence among older adults in Korea.

**Methods:**

This longitudinal study used the Korean Longitudinal Study of Aging (KLoSA) dataset from 2008 to 2018. The KLoSA is a panel survey of nationally representative samples aimed at providing data for developing socioeconomic policies for the increasing aging population in Korea. The study sample included 1967 older adults aged 65 years and over who had none or one of the chronic diseases at the baseline in 2008. Multimorbidity incidence was defined as the co-existence of two or more chronic diseases among 12 doctor-diagnosed diseases based on self-reports. Cox’s proportional hazards models were used to identify significant predictors of multimorbidity incidence over a 10-year follow-up period.

**Results:**

Among 1967 respondents (female 54.5%, mean age 72.94), 625 (31.8%) incidents of multimorbidity were reported, contributing to 47.5 incidents per 1000 people after 10 years of follow-up. Low levels of social interaction, obesity, past smoking habits, and current or past drinking habits were identified as significant predictors of multimorbidity incidence among older adults in Korea.

**Conclusions:**

This study identified older adults at high risk for multimorbidity incidence. These groups require more attention from health care providers in the course of chronic disease monitoring and management. Specific interventions and health policies to promote social interaction and a healthy lifestyle are essential to delay multimorbidity incidence. This longitudinal approach will contribute to developing preventive strategies to reduce the incidence of multimorbidity among older adults.

**Supplementary Information:**

The online version contains supplementary material available at 10.1186/s12877-022-03250-w.

## Background

By 2050, the world population of people aged 60 years or older will double to 2.1 billion, accelerating the aging rate of the population [1]. With this demographic change, multimorbidity, the co-existence of multiple health conditions, has become a global concern and has increasingly gained public health attention [2, 3]. A recent systematic review of 41 studies displayed high prevalence rates, ranging from 65% in the US to 98% in Canada, among adults aged 65 years and older [2]. According to a Korean study based on the analysis of pooled cross-sectional data based on nationally representative surveys, an overall increase of the multimorbidity prevalence in the population aged 19 and older was observed over a period of 10 years [4]. The increasing prevalence will place a substantial burden on individuals, family members, and the healthcare system in the near future [5]. Multimorbidity leads to poor health outcomes including low quality of life [6], functional decline [7], and high risk of mortality [8]. Moreover, it results in increased healthcare utilization and expenditure [4, 5]. Therefore, multimorbidity has received increased attention not only for individuals but also for healthcare systems [9].

A number of studies have investigated the prevalence and incidence of multimorbidity in various populations [10–14]. In a systematic review, the incidence rate of multimorbidity has ranged from 2.56 per 1000 person-years to 329 per 1000 person-years [15]. As part of the prevention efforts, a number of studies have focused on identifying key determinants and high-risk populations of multimorbidity across different conditions and age groups. Consistent associations have been found between multimorbidity incidence and sociodemographic characteristics, such as older age [10, 16, 17], female gender [18], lower educational attainment [16], lower income [10, 16], and living alone [16]. In lifestyle aspects, physical activities [12, 16], smoking [10, 11, 18], drinking [16], obesity [10, 11, 16], irregular involvement in food preparation [18], and inadequate dietary intake [18] have been reported as predictors or correlates of multimorbidity. Besides, multimorbidity incidence has been associated with increased cholesterol levels [11], hypertension [16], and having one chronic disease at baseline [16]. Despite the vast amount of literature on multimorbidity incidence, most of these studies focused on multimorbidity in a general population of adults, indicating that there are still gaps in our understanding regarding multimorbidity incidence in community-dwelling older adults. Furthermore, studies using a longitudinal approach are still scarce. Few longitudinal studies exist, but they used a relatively short follow-up period, ranging from 1.5 to 5 years [13, 17, 18], suggesting the need for a longer-term follow up to better understand the incidence of multimorbidity and related factors along the natural course of aging. In addition, previous longitudinal studies used a sample with a wide age range [10–14], limiting our understanding regarding key determinants of multimorbidity in the geriatric population.

Recently, there has been increasing interest in the role of psychosocial factors in contributing to multimorbidity occurrence. The Lifecourse Model of Multimorbidity Resilience demonstrates a complex set of individual, social, and environmental resources to promote multimorbidity resilience, and individual resources include psychological factors such as self-efficacy and optimism [19]. This model also suggests that a resilient integration of these resources decreases the risk of chronic diseases and improves optimal adaptation to illness. Previous studies have demonstrated a link between the presence of depressive symptoms and chronic disease burden [20–22]. For instance, in a 12-year follow-up study of working-age adults aged 50–62 in the US, individuals with depression at baseline were reported to have high risk for new onset of chronic illness, such as diabetes and heart diseases [21]. In terms of social relationships, multimorbidity was cross-sectionally related to increased loneliness [23–26] and increased social exclusion [24]. In a longitudinal study for English aging population, people without friends had higher odds of multimorbidity than those who had supportive friends, and the perception of loneliness was positively related to multimorbidity existence [27]. However, there are few longitudinal studies examining the relationship between multimorbidity and increased loneliness [25, 27] and lack of social participation [27]. Given these results, understanding the nature and extent of the association between psychosocial factors and multimorbidity is of increasing importance.

In order to tackle the increasing multimorbidity challenge as a global health concern, this study aimed to investigate the incidence rates of newly developed multimorbidity, co-existence of two or more diseases among older adults with no or one chronic disease at baseline, over a 10-year period from 2008 to 2018 using a longitudinal cohort of community-dwelling Korean older adults. We also examined the associations of sociodemographic characteristics, lifestyle, and psychosocial factors with the development of multimorbidity in this population.

## Methods

### Study design and source of data

This was a population-based, longitudinal study based on the dataset from six waves of the Korean Longitudinal Study of Aging (KLoSA). The KLoSA is an ongoing longitudinal panel survey of a nationally representative sample of individuals aged 45 years or older. The KLoSA has been conducted by the Korea Employment Information Service from 2006 to 2018 with the aim of developing socioeconomic policies for the aging population in Korea. The data obtained for this study was officially approved by Statistics Korea (Approval No. 33602) [28]. The survey included a wide range of questions asking about household background, demographic characteristics, health, employment, income and consumption, quality of life, and death [29]. KLoSA participants were recruited from the households included in the 2005 Korean Population and Housing Census. They were followed up every 2 years and surveyed by trained interviewers using a computer-assisted interviewing method. In the fifth survey in 2014, a sample of 920 participants born in 1962 and 1963 was newly added to the panel. The survey sample was randomly selected using stratified multistage probability sampling based on province and housing type [28]. At the baseline data collection in 2006, 10,254 individuals participated in the survey, and the retention rate was 77.6% with 6136 valid individuals in the 2018 survey [29].

### Study sample

**Fig. 1:**
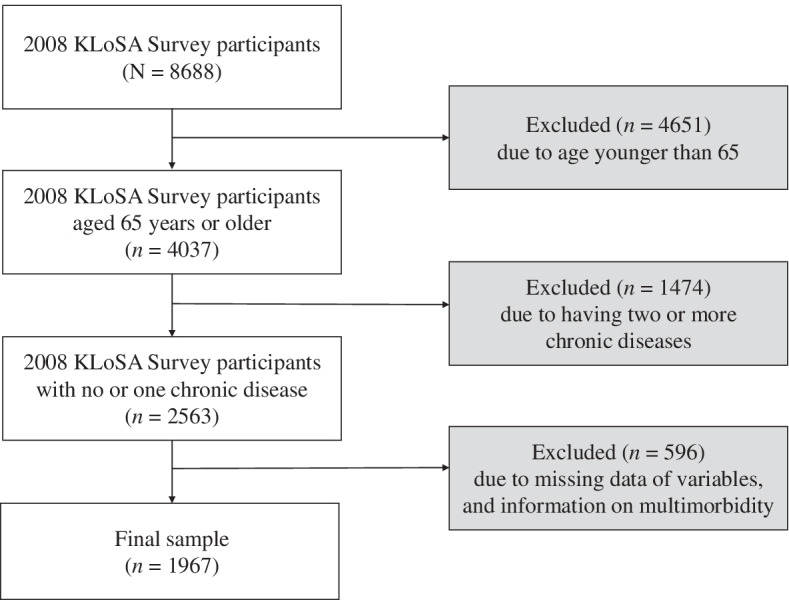
Flowchart of study samples

We set the latest data of the 2018 KLoSA as the endpoint and the 2008 KLoSA as the baseline to understand the most recent health status and track multimorbidity incidence over 10 years. Among the 8688 participants included in the 2008 KLoSA dataset, 1967 eligible subjects were included in this study. The exclusion criteria were: (1) those aged younger than 65 years at the baseline; (2) having more than two chronic diseases at the baseline; (3) no information on multimorbidity since the 2008 survey; (4) no information at the time of multimorbidity incidence since the 2008 survey; and (5) with missing data regarding all considerable variables at the baseline (Fig. 1).

### Measures

#### Dependent variable

This study used self-reported information on the year and month of 12 doctor-diagnosed morbidity conditions to assess multimorbidity (hypertension, diabetes, cancer, lung disease, liver disease, heart disease, cerebrovascular disease, arthritis or rheumatoid disease, prostate disease, digestive system disease, disc disease, and dementia). Multimorbidity in our analysis was defined as the co-existence of two or more of the conditions [30] mentioned above.

#### Independent variables

Sociodemographic information included age, gender, education level, marital status, living arrangement, working status, and income. For the analysis, age groups were categorized as young-old (65–74), old-old (75–84), and oldest-old (85 and over). Gender was dichotomized as either male or female. The education level was classified into three groups: (1) no formal education, (2) elementary school, and (3) middle school or above. Marital status was categorized as (1) married and (2) single or other according to whether an individual had a partner or a significant other. Living arrangements were classified into two groups: single-person households and multi-person households. Lastly, working status was dichotomized as none or working. The annual personal income of the previous year was obtained in units of 10,000 Korean Won.

For lifestyle factors, body mass index (BMI; weight in kilograms divided by height in meters squared) was measured as physical health. BMI was categorized as (1) underweight (< 18.5 kg/m^2^), (2) normal (≥ 18.5 and < 23 kg/m^2^), (3) overweight (≥ 23 and < 25 kg/ m^2^), and (4) obese (≥ 25 kg/ m^2^) at the baseline. This categorization was based on the agreed cutoff for Asia–Pacific countries [31]. Health behaviors included physical activities, smoking, and drinking habits. Participants were asked whether they performed any type of physical activity at least once a week. Physical activity was dichotomized as physically inactive (not performing any physical activity) and physically active (doing regular exercise at least once a week). The categorization was based on the fact that even mild physical activity has been associated with a lower risk of cardiovascular disease and mortality among older adults [32]. Smoking behaviors were categorized as (1) non-smokers (having never smoked more than 5 packs in their whole life and no smoking at the time of the interview), (2) past smokers (no smoking at the time of the interview, but having smoked more than 5 packs in their whole life), and (3) current smokers (smoking at the time of the interview). Similarly, drinking behaviors were categorized as (1) non-drinkers (no alcohol consumption at the time of the last and current interviews), (2) past drinkers (alcohol consumption at the time of last interview, but no alcohol consumption at the current interview), and (3) current drinkers (alcohol consumption at the time of the interview).

For psychosocial factors, depressive symptoms were measured using 10 items retrieved from the Korean 20-item version of the Center for Epidemiological Studies-Depression Scale (CES-D) [33] included in the 2008 KLoSA survey. The CES-D was used to assess depressive symptoms from the previous week [34]. Possible scores range from 0 to 10, with higher scores indicating a higher level of depression. The social network was measured by two variables: participation in social meetings and the level of social interaction. The social meeting participation was dichotomized as (1) no social participation and (2) participation in more than one social meeting. Examples of social meetings include religious gatherings, meeting friends, and volunteering. The level of social interaction was the frequency of contact with friends, relatives, or neighbors who live close to the participant for the past year. This item had three response options: low (less than five or six times a year), moderate (once or twice a month), and high (more than twice or three times a week).

### Statistical analysis

Descriptive statistics were used to describe the baseline characteristics of the sample using means with standard deviations (*SD*) or frequencies with proportions (%). Independent t-tests for continuous variables and chi-square tests for categorical variables were performed to compare the multimorbidity group to the non-multimorbidity group, as well as individuals with no chronic conditions and those with one chronic condition reported at baseline. Multimorbidity incidence rates during follow-up were estimated by Kaplan–Meier methods. Crude incidence rates of multimorbidity were calculated by the number of newly doctor-diagnosed second disease per 1000 person-years. The Cox's proportional hazards models were used to examine the association between the potential predictors and the multimorbidity incidence during 10-year follow-up using the baseline data of major sociodemographic characteristics, lifestyle, and psychosocial factors. The selection of the modeling technique was based on the intention to help prevent multimorbidity incidence in advance by identifying the predictors according to the baseline value, regardless of the time-variant variables. All significant variables based on univariate analysis and confounders, including age, gender, and number of chronic diseases at the baseline, were used to estimate adjusted hazard ratios (aHR). The risk of a variable was presented as an aHR with a corresponding 95% confidence interval (CI). They reported a significance level of 0.05. All analyses were performed using SAS 9.4 (SAS Institute, Cary, NC, USA).

## Results

### Baseline characteristics of participants

We included 1967 older adults with no or one chronic disease at the baseline. The mean age of the sample was 72.94 years (*SD* = 6.42), and 54.5% of the sample were female. About 70% of respondents had either no formal education (33.6%) or only attended elementary school (34.4%). Most subjects reported living with other household members (85.0%). Almost half of the participants had normal BMI (51.5%) at baseline. This was followed by participants who were overweight (26.1%), obese (15.5%), and underweight (6.9%). On average, the number of depressive symptoms reported was 4.16 (*SD* = 2.96). Approximately 70% of them participated in some types of social meetings, and 69.2% of them reported that they had social interaction with friends, relatives, or neighbors at least two or three times a week (Table 1). Baseline characteristics of the sample were stratified according to the number of diseases reported at baseline: none (*n* = 927) and one disease (*n* = 1040) (Additional file 1 Table S1).

**Table 1:** Baseline characteristics of the study population, by 10-year multimorbidity incidence (*N* = 1967)

Variables		Mean ± *SD* or *n* (%)			*P*-value^*^
		All participants (*N* = 1967)	Multimorbidity incidence		
			Yes (*n* = 625)	No (*n* = 1342)	
**Sociodemographic characteristics**					
Age		72.94 ± 6.42	71.98 ± 5.36	73.38 ± 6.82	< 0.001
	65–74	1311 (66.6)	446 (71.4)	865 (64.4)	< 0.001
	75–84	527 (26.8)	161 (25.7)	366 (27.3)	
	85–	129 (6.6)	18 (2.9)	111 (8.3)	
Gender	Male	894 (45.5)	299 (47.8)	595 (44.3)	0.146
	Female	1073 (54.5)	326 (52.2)	747 (55.7)	
Education level	No formal education	660 (33.6)	184 (29.4)	476 (35.5)	0.021
	Elementary school	677 (34.4)	221 (35.4)	456 (34.0)	
	Middle school or above	630 (32.0)	220 (35.2)	410 (30.5)	
Marital status	Married	1335 (67.9)	454 (72.6)	881 (65.6)	0.002
	Single or other	632 (32.1)	171 (27.4)	461 (34.4)	
Living arrangement	Single-person households	296 (15.0)	84 (13.4)	212 (15.8)	0.173
	Multiple-person households	1671 (85.0)	541 (86.6)	1130 (84.2)	
Working status	Working	539 (27.4)	182 (29.1)	357 (26.6)	0.244
	None	1428 (72.6)	443 (70.9)	985 (73.4)	
Annual personal income (10,000 Korean Won)		694.48 ± 885.02	653.6 2 ± 751.50	713.50 ± 940.42	0.130
**Lifestyle**					
BMI	Underweight	136 (6.9)	30 (4.8)	106 (7.9)	< 0.001
	Normal	1013 (51.5)	291 (46.6)	722 (53.8)	
	Overweight	513 (26.1)	170 (27.2)	343 (25.6)	
	Obese	305 (15.5)	134 (21.4)	171 (12.7)	
Physical activity	Yes	612 (31.1)	215 (34.4)	397 (29.6)	0.032
	No	1355 (68.9)	410 (65.6)	945 (70.4)	
Smoking	Current	330 (16.8)	100 (16.0)	230 (17.1)	0.003
	Past	278 (14.1)	113 (18.1)	165 (12.3)	
	Never	1359 (69.1)	412 (65.9)	947 (70.6)	
Drinking	Current	607 (30.9)	211 (33.7)	396 (29.5)	< 0.001
	Past	219 (11.1)	88 (14.1)	131 (9.8)	
	Never	1141 (58.0)	326 (52.2)	815 (60.7)	
**Psychosocial factors**					
Depressive symptoms (0–10)		4.16 ± 2.96	4.24 ± 2.93	4.13 ± 2.97	0.441
Participation in social meeting	Yes	1339 (68.1)	418 (66.9)	921 (68.6)	0.439
	No	628 (31.9)	207 (33.1)	421 (31.4)	
Level of social interaction	High	1360 (69.2)	407 (65.1)	953 (71.0)	0.003
	Moderate	327 (16.6)	105 (16.8)	222 (16.5)	
	Low	280 (14.2)	113 (18.1)	167 (12.5)	
**No. of chronic diseases**					
0		927 (47.1)	163 (26.1)	764 (56.9)	< 0.001
1		1040 (52.9)	462 (73.9)	578 (43.1)	

*BMI* body mass index, *No.* number, *SD* standard deviation

^*^Independent t-test or chi-square test

### The incidence rates of multimorbidity

**Fig. 2:**
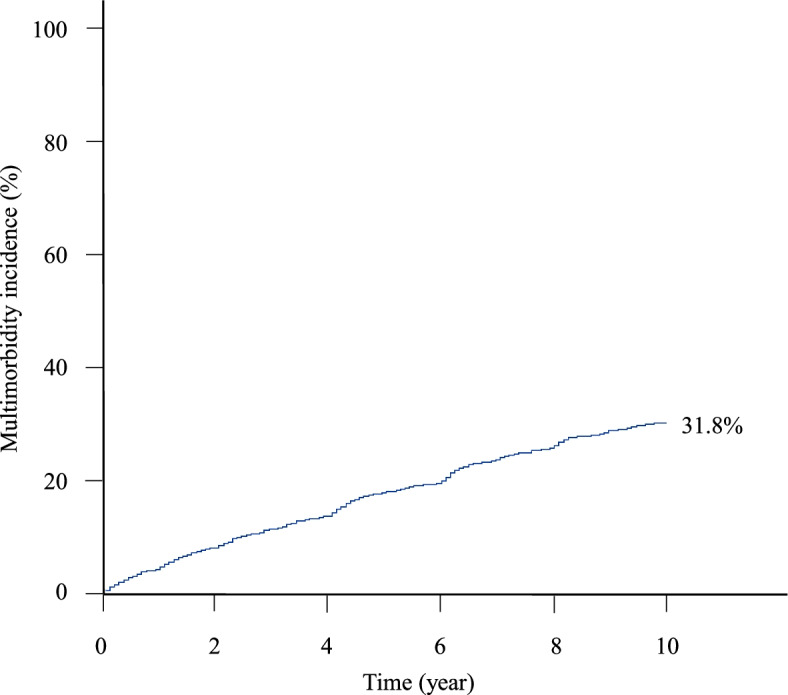
Cumulative incidence of multimorbidity for 10-year follow-up

Over a 10-year follow-up period, 625 participants developed multimorbidity (Table 1). A total of 1967 participants contributed 47.5 incidents per 1000 person-years of follow-up in the 625 cases of multimorbidity. Moreover, a 31.8% cumulative incidence rate was estimated (Fig. 2). The unadjusted comparison showed statistically significant differences in age, education level, marital status, BMI, physical activity, smoking and drinking habits, level of social interaction, and the number of chronic diseases at baseline between the multimorbidity and non-multimorbidity groups (Table 1).

### Predictors of multimorbidity

When each factor was assessed independently (Table 2), past drinking was the strongest predictor (HR: 1.73, 95% CI: 1.37–2.19) of multimorbidity incidence during the 10-year follow-up period. Also, no participation in social meetings (HR: 1.19, 95% CI: 1.01–1.41), low social interaction level (HR: 1.61, 95% CI: 1.31–1.99), obesity (HR: 1.65, 95% CI: 1.34–2.02), past smoking (HR: 1.55, 95% CI: 1.26–1.91), and current drinking (HR: 1.25, 95% CI: 1.05–1.49) significantly increased the risk of multimorbidity incidence.

**Table 2:** Hazard ratios (HRs) for multimorbidity incidence (*N* = 1967)

Variables		Univariate model	Multivariate model^a^
**Lifestyle**			
BMI	Normal	Reference	Reference
	Underweight	0.96 (0.66–1.39)	0.95 (0.65–1.39)
	Overweight	1.16 (0.96–1.41)	1.07 (0.88–1.29)
	Obese	1.65 (1.34–2.02)	1.51 (1.22–1.86)
Physical activity	Yes	Reference	
	No	0.92 (0.78–1.08)	–
Smoking	Never	Reference	Reference
	Past	1.55 (1.26–1.91)	1.31 (1.03–1.70)
	Current	1.04 (0.84–1.30)	1.08 (0.83–1.40)
Drinking	Never	Reference	Reference
	Past	1.73 (1.37–2.19)	1.55 (1.18–2.05)
	Current	1.25 (1.05–1.49)	1.38 (1.11–1.72)
**Psychosocial factors**			
Depressive symptoms		1.03 (1.00–1.05)	–
Participation in social meeting	Yes	Reference	
	No	1.19 (1.01–1.41)	1.09 (0.91–1.30)
Level of social interaction	High	Reference	Reference
	Moderate	1.09 (0.88–1.35)	1.05 (0.84–1.30)
	Low	1.61 (1.31–1.99)	1.72 (1.38–2.14)

*BMI* body mass index, *CI* confidence interval

Values are HR (95% CI)

^a^Adjusted by age, gender and number of chronic diseases at the baseline

In a multivariate model adjusted for age, gender, and the number of chronic diseases at baseline, a low level of social interactions was the most associated with a higher incidence of multimorbidity (aHR: 1.72, 95% CI: 1.38–2.14) compared with the reference group. Drinking status was a significant predictor of multimorbidity incidence, increasing the risk by 55% for past drinkers (aHR: 1.55, 95% CI: 1.18–2.05) and 38% for current drinkers (aHR: 1.38, 95% CI: 1.11–1.72). Furthermore, obesity increased the multimorbidity risk by 51% (aHR: 1.51, 95% CI: 1.22–1.86) compared with the normal BMI group, and past smoking was associated with the hazard of multimorbidity incidence, reporting 31% of the risk (aHR: 1.31, 95% CI: 1.03–1.70). The existence of social meetings was not associated with a risk of multimorbidity in the multivariate model.

## Discussion

This study provides important information about the incidence of multimorbidity and the associated factors in the geriatric population, which has been a limitation of previous studies [10–13]. Using data from a nationally representative cohort of Korean older adults, we defined multimorbidity as the co-existence of two or more chronic diseases to identify high-risk groups for multimorbidity among adults aged 65 years and older. The multimorbidity incidence rate was 47.5 incidents per 1000 person-years in the Korean older adult population from 2008 to 2018, which was lower than that of a New Zealand study reporting 68.5 incidents per 1000 person-years of multimorbidity incidence over 10 years of follow-up among adults aged 55–70 [16], and 59 incidents per 1000 person-years among adults aged 50 years and older over 5 years of follow-up in the United Kingdom [13]. Moreover, the current study confirmed a cumulative multimorbidity incidence rate of 31.8%. This rate is lower than the 54.6% of a Swedish study on adults aged 78 years and older for 3-year follow-up [17]. These results would be explained by the differences in demographic characteristics as well as in multimorbidity measurement or definitions. For example, participants in the current study had a mean age of 72.9 years, which was lower than those aged 78 years and older (mean age 83.9) in a Swedish study [17]. In addition, multimorbidity was measured with self-reported information on diseases diagnosed by doctors in the current study, while the latter study [17] examined multimorbidity with data from clinical examination or laboratory testing. These are in line with the results of previous studies that showed older age was a significant predictor of multimorbidity incidence [10, 16] and self-reported multimorbidity was underestimated [35]. Further, our study reported a lower incidence rate based on information on 12 chronic conditions, compared to an English study including 18 doctor-diagnosed chronic diseases based on self-reported data [13]. This result is supported by the previous study reporting the more diseases included, the higher the prevalence of multimorbidity is reported [36].

There has been inconsistency in the definition and measurement of multimorbidity in peer-reviewed studies, making it challenging to compare findings on the incidence and risk factors of multimorbidity across populations. In response to this concern, it has been suggested to include the most prevalent chronic diseases in a targeted population because the list of conditions assessed would influence the prevalence estimate [3, 37]. In addition, the need for a new definition using a different cutoff (the number of diseases included) has been recently raised and debated [37] to identify the population with a high level of demand. Fortin et al. [3] reported that when the cutoff for multimorbidity was set to two, the prevalence of multimorbidity showed an ‘S’ shaped curve with a stagnant pattern for older ages. However, when a definition with three or more diseases was used, it showed a more linear pattern of multimorbidity prevalence with greater differentiation according to age. Another study also reported that defining multimorbidity as a co-existence of two or more chronic diseases resulted in a large portion of multimorbidity among older adults with a lack of specificity to identify groups with higher health needs [37]. Therefore, defining multimorbidity as the co-existence of three or more chronic conditions might be a better measure to provide more specificity and greater differentiation in the older adult population [3, 37]. Further inquiry will be necessary to determine a reasonable set of diseases and a cutoff for multimorbidity based on the characteristics of a target population.

Among the identified risk factors, the level of social interaction was the most significant factor related to multimorbidity incidence in Korean older adults. This result is in line with the previous study that confirmed the significant relationship between social support and multimorbidity prevalence [27]. Several studies demonstrated that supportive social relationships increased the likelihood of survival of people with multimorbidity [38]. Inadequate levels of perceived social relationships and loneliness are related to negative health outcomes, including a poor quality of life [39]. Social interaction may act through two mechanisms [40]. First, individuals can obtain information from a network of social relationships regarding how to modify their lifestyle and learn about useful resources for self-management of chronic conditions, including health information [41]. Second, social interaction has a direct effect on individuals’ ability to relieve their stress [42]. Through interactions with others, individuals can improve their experiences of meaningfulness in everyday life [43], emotional well-being [44], feeling of being valued, and sense of belonging [45]. Given these positive effects, promoting social interactions can be a protective strategy against multimorbidity incidence among older adults. It is well known that older adults who are socially isolated and feel they do not belong in society have negative health outcomes [46]. Furthermore, a small social network is associated with a higher rate of heart and lung disease diagnosis [47] and a lower level of quality of life [48] among older adults. While studies have examined the association between multimorbidity and social aspects of health, including social isolation and loneliness [24, 26], the relationship between them is not fully understood. Further studies are necessary to identify a group of older adults whose social activities are limited and/or feel lonely, to identify physical and psychosocial pathways linking social isolation to multimorbidity.

We found that obesity had a 1.51 times higher risk of multimorbidity incidence than those with normal weight in an adjusted model. This is consistent with previous cross-sectional [49, 50] and longitudinal studies [10, 11, 16], which reported a significant association between obesity and multimorbidity prevalence or incidence. The population with obesity has tripled over decades worldwide in the general population [51]. Evidence suggests that obesity is associated with non-communicable diseases, including cardiovascular disease, diabetes, musculoskeletal disease, and some cancers [51], affecting almost all body systems [52]. On the other hand, some studies have confirmed that low body weight in older adults is related to negative outcomes such as higher mortality, while being overweight may be a protective factor [53, 54], although low weight was not associated with a hazard of multimorbidity in the current study. For these reasons, further studies are necessary to examine the role of BMI control in the onset of multimorbidity in older adults and to identify an appropriate level of target weight to improve health outcomes. Additionally, our findings should be understood in consideration of ethnicity since the current study applied BMI standards for the Asian population [31].

We found that drinking and smoking habits were significant predictors of multimorbidity’s onset. Previous studies reported inconsistent findings regarding the effect of different types of unhealthy behaviors on the onset of multimorbidity. One study showed that current smoking status increased the risk of multimorbidity’s onset in adults with disease-free or comorbid diabetes at the baseline [11]. In a Malaysian study of adults aged 60 years and older, current smoking increased multimorbidity incidence after 1.5 years of follow-up for those who had no chronic disease at baseline [18]. A 3-year follow-up Swedish study reported that neither smoking nor drinking were significantly associated with multimorbidity incidence [17]. Additionally, an English study of adults aged 50 years or older followed up for 5 years with a focus on unhealthy lifestyle factors found that smoking and excessive alcohol consumption were not found to increase the risk of multimorbidity incidence individually [13]. It was also reported that two or more unhealthy behaviors were significantly related to multimorbidity incidence, and any combination of more than two unhealthy behaviors was likely to increase the risk of multimorbidity incidence [13]. In contrast, a New Zealand study of adults aged 55–70 followed up for 10 years identified regular alcohol consumption as a protective predictor of multimorbidity incidence. Moreover, it found that there was no association between smoking and the onset of multimorbidity [16]. Despite the mixed findings, these studies indicate the importance of public health efforts to focus on primary prevention of risks for multimorbidity incidence by encouraging the practice of healthy lifestyle habits in the population. This is because any combination of unhealthy behaviors can increase the risk of multimorbidity and also because health behaviors can be a modifiable target. Future research should consider the strict classification of drinking and smoking on the amount and intensity of specific population groups to determine the causal relationship and to establish effective strategies.

In the current study, depressive symptoms were not associated with multimorbidity incidence in both univariate and multivariate models, different from the framework of the Lifecourse Model of Multimorbidity Resilience [19] and previous studies [21, 55]. However, high depression prevalence is one of the most critical challenges for aging societies [56], and the risk of disability and mortality has been reported to be higher when multimorbidity and depression coexist [57, 58]. As suggested by the bidirectional relationship between multimorbidity and depression [56, 59], further studies should be undertaken to investigate the association between depression and multimorbidity. In addition, depression prevention is essential for improving both physical and psychological health outcomes among older adults.

### Strengths and limitations

This study is a longitudinal study with a 10-year follow-up of community-dwelling older adults. It used a nationally representative, prospective cohort of older adults, providing evidence on multimorbidity incidence and its predictors in South Korea. The major strength of the current study is that it examined psychosocial factors associated with the onset of multimorbidity, which has been less investigated in previous studies. Our findings indicate a need for establishing health policies focused on improving social interactions among older adults to lower the incidence of multimorbidity. These are important steps to manage a population at a high risk of worsening health conditions.

This study has several limitations. First, chronic disease diagnosis and the time of diagnosis in the KLoSA are self-reported, resulting in potential bias in the results. For instance, the incidence rates would be underestimated because self-reported prevalence estimates were found to be lower than when physical exam-based data were collected [35]. In addition, there may be a possibility that time-related information on doctor-diagnosis has been misestimated due to recall bias or memory issues in older adults. Second, because of the limitations in the original dataset, we were not able to include a few chronic diseases prevalent in the geriatric population, such as chronic kidney disease, osteoarthrosis, and Parkinson’s disease. Further, some diseases might be counted as one entity (e.g. heart disease). This might have led to an underestimation of multimorbidity incidence in the current study. Third, we did not consider the burden of an increasing number of chronic diseases or the severity of each chronic condition. This would lead to covering up the impact of health problems for older adults. Future studies need to consider different approaches to measuring multimorbidity, such as the total number of chronic diseases or weighting [60] to identify at-risk groups unless otherwise detected. Finally, there may have been a bias in the calculation of the incidence rate since we excluded those with no data about the diagnosis timing.

## Conclusions

This study indicated that a significant number of Korean older adults have developed multiple chronic diseases over 10 years. The current study advanced our current knowledge about multimorbidity and its determinants in older adults, such as lifestyle and psychosocial factors. All of the risk factors identified in our study are modifiable, suggesting important implications for future population health strategies. Health policies are required to tackle unhealthy behaviors and lack of social participation and provide system-wide primary care to screen at-risk individuals. In future research, it is important to incorporate these findings into the development of programs and services for social engagement as well as health promotion targeting unhealthy behaviors in community-dwelling older adults.

## Supplementary Information

**Additional file 1:** **Table S1. **Baseline characteristics of the study population according to the number of chronic diseases (*N* = 1967).

## Data Availability

The data analyzed in the current study are available at: https://survey.keis.or.kr/eng/klosa/databoard/List.jsp

## Abbreviations

aHR: Adjusted hazard ratio
BMI: Body mass index
CES-D: Center for Epidemiological Studies-Depression Scale
CI: Confidence interval
KLoSA: Korean Longitudinal Study of Aging
SD: Standard deviation
