# Supplementary material for: Incidence and predictors of multimorbidity among older adults in Korea: a 10-year cohort study
Source: BMC Geriatr. 2022 Jul 7;22:565. doi: 10.1186/s12877-022-03250-w (PMC9264523; doi:10.1186/s12877-022-03250-w)
Supplement: Supplementary file 1 — Additional file 1: Table S1. Baseline characteristics of the study population according to the number of chronic diseases (N = 1967). [file 12877_2022_3250_MOESM1_ESM.docx]

**Table S1. Baseline characteristics of the study population according to the number of chronic diseases (N = 1967)**

| **Variables** | | | | **Mean ± *SD* or *n* (%)** | | | ***P*-value**^*^ |
| --- | --- | --- | --- | --- | --- | --- | --- |
|  |  |  |  | **Total**  **(N = 1967)** | **none**  **(*n* = 927)** | **one**  **(*n* = 1040)** |  |
| **Socioeconomic characteristics** | | | |  |  |  |  |
| Age | |  | 72.94 **±** 6.42 | 72.78 **±** 6.67 | 73.07 **±** 6.20 | 0.314 |  |
|  | 65–74 | | 1311 (66.6) | 642 (69.3) | 669 (64.3) | 0.003 |  |
|  | 75–84 | | 527 (26.8) | 216 (23.3) | 311 (29.9) |  |  |
|  | 85– | | 129 (6.6) | 69 (7.4) | 60 (5.8) |  |  |
| Gender | Male | | 894 (45.5) | 463 (50.0) | 431 (41.4) | < 0.001 |  |
|  | Female | | 1073 (54.5) | 464 (50.0) | 609 (58.6) |  |  |
| Education level | No formal education | | 660 (33.6) | 310 (33.4) | 350 (33.7) | 0.883 |  |
|  | Elementary school | | 677 (34.4) | 324 (35.0) | 353 (33.9) |  |  |
|  | Middle school or above | | 630 (32.0) | 293 (31.6) | 337 (32.4) |  |  |
| Marital status | Married | | 1335 (67.9) | 642 (69.3) | 693 (66.6) | 0.214 |  |
|  | Single or other | | 632 (32.1) | 285 (30.7) | 347 (33.4) |  |  |
| Living arrangement | Single-person households | | 296 (15.1) | 129 (13.9) | 167 (16.1) | 0.185 |  |
|  | Multiple-person households | | 1671 (85.0) | 798 (86.1) | 873 (83.9) |  |  |
| Working status | Working | | 539 (27.4) | 292 (31.5) | 247 (23.7) | < 0.001 |  |
|  | None | | 1428 (72.6) | 635 (68.5) | 793 (76.3) |  |  |
| Annual personal income (10,000 Korean Won) | | | 694.48 **±** 885.02 | 767.70 **±** 921.20 | 629.21 **±** 846.61 | < 0.001 |  |
|  | | |  |  |  |  |  |
| **Lifestyle** | | |  |  |  |  |  |
| BMI | | Underweight | | 136 (6.9) | 73 (7.9) | 63 (6.0) | < 0.001 |
|  | | Normal | | 1013 (51.5) | 510 (55.0) | 503 (48.4) |  |
|  | | Overweight | | 513 (26.1) | 232 (25.0) | 281 (27.0) |  |
|  | | Obese | | 305 (15.5) | 112 (12.1) | 193 (18.6) |  |
| Physical activity | Yes | | 612 (31.1) | 276 (29.8) | 336 (32.3) | 0.226 |  |
|  | No | | 1355 (68.9) | 651 (70.2) | 704 (67.7) |  |  |
| Smoking | Current | | 330 (16.8) | 188 (20.3) | 142 (13.6) | < 0.001 |  |
|  | Past | | 278 (14.1) | 121 (13.0) | 157 (15.1) |  |  |
|  | Never | | 1359 (69.1) | 618 (66.7) | 741 (71.3) |  |  |
| Drinking | Current | | 607 (30.9) | 322 (34.7) | 285 (27.4) | < 0.001 |  |
|  | | Past | | 219 (11.1) | 88 (9.5) | 131 (12.6) |  |
|  | | Never | | 1141 (58.0) | 517 (55.8) | 624 (60.0) |  |
|  | | |  |  |  |  |  |
| **Psychosocial factors** | | |  |  |  |  |  |
| Depressive symptoms (0–10) | | | 4.16 **±** 2.96 | 3.95 **±** 2.89 | 4.35 **±** 3.00 | 0.003 |  |
| Participation in social meeting | Yes | | 1339 (68.1) | 654 (70.5) | 685 (65.9) | 0.026 |  |
|  | No | | 628 (31.9) | 273 (29.5) | 355 (34.1) |  |  |
| Level of social interaction | High | | 1360 (69.2) | 644 (69.5) | 716 (68.9) | 0.877 |  |
|  | Moderate | | 327 (16.6) | 155 (16.7) | 172 (16.5) |  |  |
|  | Low | | 280 (14.2) | 128 (13.8) | 152 (14.6) |  |  |

*BMI* body mass index, *SD* standard deviation

^*^Independent t-test or chi-square test
